# Comparing Crowdsourcing and Friendsourcing: A Social Media-Based Feasibility Study to Support Alzheimer Disease Caregivers

**DOI:** 10.2196/resprot.6904

**Published:** 2017-04-10

**Authors:** Daniel Robert Bateman, Erin Brady, David Wilkerson, Eun-Hye Yi, Yamini Karanam, Christopher M Callahan

**Affiliations:** ^1^ Indiana University Center for Aging Research Indianapolis, IN United States; ^2^ Department of Psychiatry Indiana University School of Medicine Indianapolis, IN United States; ^3^ Regenstrief Institute Indianapolis, IN United States; ^4^ Indiana Alzheimer Disease Center Indianapolis, IN United States; ^5^ Department of Human-Centered Computing School of Informatics and Computing Indiana University Purdue University Indianapolis Indianapolis, IN United States; ^6^ School of Social Work Indiana University Indianapolis, IN United States; ^7^ Department of Medicine Indiana University School of Medicine Indianapolis, IN United States

**Keywords:** Alzheimer disease, Alzheimer disease and related dementias, caregivers, mobile health, social media, crowdsourcing, friendsourcing, emotional support, informational support, online support

## Abstract

**Background:**

In the United States, over 15 million informal caregivers provide unpaid care to people with Alzheimer disease (AD). Compared with others in their age group, AD caregivers have higher rates of stress, and medical and psychiatric illnesses. Psychosocial interventions improve the health of caregivers. However, constraints of time, distance, and availability inhibit the use of these services. Newer online technologies, such as social media, online groups, friendsourcing, and crowdsourcing, present alternative methods of delivering support. However, limited work has been done in this area with caregivers.

**Objective:**

The primary aims of this study were to determine (1) the feasibility of innovating peer support group work delivered through social media with friendsourcing, (2) whether the intervention provides an acceptable method for AD caregivers to obtain support, and (3) whether caregiver outcomes were affected by the intervention. A Facebook app provided support to AD caregivers through collecting friendsourced answers to caregiver questions from participants’ social networks. The study’s secondary aim was to descriptively compare friendsourced answers versus crowdsourced answers.

**Methods:**

We recruited AD caregivers online to participate in a 6-week-long asynchronous, online, closed group on Facebook, where caregivers received support through moderator prompts, group member interactions, and friendsourced answers to caregiver questions. We surveyed and interviewed participants before and after the online group to assess their needs, views on technology, and experience with the intervention. Caregiver questions were pushed automatically to the participants’ Facebook News Feed, allowing participants’ Facebook friends to see and post answers to the caregiver questions (Friendsourced answers). Of these caregiver questions, 2 were pushed to crowdsource workers through the Amazon Mechanical Turk platform. We descriptively compared characteristics of these crowdsourced answers with the friendsourced answers.

**Results:**

In total, 6 AD caregivers completed the initial online survey and semistructured telephone interview. Of these, 4 AD caregivers agreed to participate in the online Facebook closed group activity portion of the study. Friendsourcing and crowdsourcing answers to caregiver questions had similar rates of acceptability as rated by content experts: 90% (27/30) and 100% (45/45), respectively. Rates of emotional support and informational support for both groups of answers appeared to trend with the type of support emphasized in the caregiver question (emotional vs informational support question). Friendsourced answers included more shared experiences (20/30, 67%) than did crowdsourced answers (4/45, 9%).

**Conclusions:**

We found an asynchronous, online, closed group on Facebook to be generally acceptable as a means to deliver support to caregivers of people with AD. This pilot is too small to make judgments on effectiveness; however, results trended toward an improvement in caregivers’ self-efficacy, sense of support, and perceived stress, but these results were not statistically significant. Both friendsourced and crowdsourced answers may be an acceptable way to provide informational and emotional support to caregivers of people with AD.

## Introduction

Studies predict that the worldwide prevalence of dementia will reach 48.1 million people by the year 2020 [1]. Alzheimer disease (AD) accounts for the majority of cases of dementia in the United States [2]. Unlike for many other diseases, to date there are no disease-modifying agents to slow down or stop the progression of AD. Until disease-modifying agents become available, psychosocial and psychoeducational interventions remain the intervention of choice for addressing treatment needs of patients and caregivers. Informal, unpaid caregivers deliver the majority of care received by people with AD [3]. Estimates for 2013 put the value of this informal, unpaid care for people with AD and other dementias in the United States at US $470 billion [3]. Fiscal estimates do not account for the mental and physical health burdens endured by many AD caregivers. Besides the inherent detriment to caregivers, higher caregiver burden and lower caregiver subjective health ratings adversely affect patients with AD. Higher caregiver burden results in earlier nursing home placement, while lower caregiver subjective health is associated with higher mortality in people with AD [4]. Prior work showed that AD caregivers receive health benefits from psychosocial and psychoeducational interventions [5]. Yet many caregivers’ needs remain unmet due to multiple barriers, including time and distance. Social media offers an opportunity to provide support to AD caregivers while overcoming some of these barriers [5].

Caregivers have a higher incidence of mental illness and benefit from emotional support. These mental illnesses include anxiety [6-8], depression [6,9-15], poor sleep quality [7,8], and substance abuse or dependence [16]. Caregiving also affects the work force, with caregivers displaying greater absenteeism [6,7], higher rates of poor physical health [17-19], and higher mortality [20,21]. Studies show that strategies of problem-focused coping, acceptance, and social-emotional support improve caregiver mental health and depression [3,22-26].

Caregivers receive emotional and informational support from many sources, but these existing sources have limitations. In-person social support groups can meet caregiver support needs including emotional support and self-appraisal [27], but they present limitations based on the logistical issues of scheduling, traveling, and finding alternative supervision for the person with dementia while the caregiver is absent. Membership in online health communities can address logistical barriers to service utilization, and caregivers often seek emotional and informational support from their peers [28]. However, cautions about the reliability of health care information being shared [29], the tendency of participants to lurk by browsing rather than contributing content [30,31], and participation inequalities [32] point to limitations that interfere with the ability of online communities to support caregivers in the process of coping.

Other systems may provide individualized support for the caregiver, supplementing the benefits of traditional forums. However, any technological system designed to provide just-in-time personalized support to caregivers requires a large number of people who are online and available to respond to their questions. Systems that connect a caregiver to a clinician or trained social worker are unlikely to scale well, due to the limited size of the clinician population. For this study, we investigated *crowdsourcing* and *friendsourcing* as alternatives to soliciting appropriate and supportive feedback to questions from caregivers.

Crowdsourcing is a way to leverage remote workers to perform small tasks, either for financial compensation or on a voluntary basis [33]. In crowdsourced systems, a task is broken up into parts and distributed to remote workers, who can complete the sections in parallel or build on the work of others. The answers provided by these workers can be aggregated and used to create systems that leverage human intelligence in novel ways. Crowdsourcing systems have been used to provide emotional support to individuals by collecting empathetic responses or cognitive reappraisals of stressful situations [34]. While online forums are used by many to seek information, crowdsourcing can be a more efficient and reliable way of seeking information. Drawbacks of traditional crowdsourcing include financial costs and variable quality of answers, depending on the expertise and experience of the crowd workers.

Friendsourcing is a paradigm that combines social media information seeking with crowdsourcing [35]. In friendsourcing, individuals use their friends and contacts online as a resource for crowdsourcing information or help. While friendsourcing can be used to identify specific information that only your network would know [35], as people gain stronger trust in the information their friends or families provide, they often leverage these social networks to seek information that might be available to them in other places [36]. Prior work has shown that as many as 50% of social media users have friendsourced a question to their online networks before [36], and that social media can have significant impacts on health-related behavior change and well-being [37,38]. There are often benefits to providing this information as well. People answering questions have improved self-efficacy, build a sense of reciprocity with the question asker, and appreciate the opportunity to show off their expertise [36,39].

Online peer support activities can help AD caregivers reduce the stress of caregiving and enhance hands-on knowledge and self-efficacy. A common model of online peer support involves the use of online discussion forums. Drawbacks of the online discussion forum model include low user content contribution and limited efficiency for accessing correct information.

We designed this pilot study to explore alternative online methods to address caregiver needs through online peer support. This research conducted by an interdisciplinary team aimed to overcome previously mentioned drawbacks by adapting friendsourcing, within a closed Facebook group. To our knowledge, our work is the first to use friendsourcing to provide support to AD caregivers. The underpinning theory of this intervention is that social support moderates caregiver burden and stress. We hypothesized that the study intervention would be acceptable to AD caregivers as a method for obtaining online support, that friendsourced and crowdsourced answers to caregiver questions would be of high-quality content, and that friendsourced answers would have higher rates of shared experiences in comparison with crowdsourced answers.

The contributions of this study include (1) an assessment of the feasibility of online support delivery to AD caregivers through social media and friendsourcing, (2) an evaluation and comparison of friendsourced and crowdsourced answers to caregiver questions in terms of content quality, acceptability, and rates of shared experiences, and (3) suggestions for future research directions for online support delivery to AD caregivers through social media.

## Methods

### Study Participants and Recruitment

Potential participants were nonpaid family caregivers of people with AD. We distributed a recruiting advertisement across the United States through organizational webpages, including the Alzheimer’s Association webpage and newsletter, Indiana University Purdue University Indianapolis’s online newsletter *News at IUPUI*, radio interviews, and social media, including Twitter (Twitter, Inc, San Francisco, CA, USA) and Facebook (Facebook, Inc, Menlo Park, CA, USA), from July through September 2016. Potential participants gained access to the study through the study website. The website provided study information, inclusion and exclusion criteria, links to the online survey, and a PDF of the informed consent form. This study was approved by the Indiana University Institutional Review Board (# 160317338) through expedited review.

Study inclusion criteria for participation were as follows: the participant must (1) be 18 years or older, (2) live in the United States, (3) be able to read, comprehend, and write in English, (4) be the caregiver of an individual with AD who lives at home with the caregiver, (5) provide at least 8 hours of caregiving for the person with AD per week, (6) have a Facebook account with at least 40 Facebook friends, (7) have posted or commented on Facebook on average at least twice per week for the past month, (8) have ready access to the Internet, and (9) agree to give his or her informed consent to participate in this research. Exclusion criteria were a psychiatric hospitalization or suicide attempt in the past year. We chose age 18 years as the cutoff so that all participants were legal adults. The requirement of 40 Facebook friends was arbitrarily chosen as a cutoff to indicate the participant had a preexisting online social network before joining the study.

### Design

This study used a pretest-posttest design with mixed methods. We sequentially allotted participating nonpaid family caregivers of people with AD to 3 closed Facebook groups to receive the intervention over the course of 6 weeks (September 29, 2016 to October 10, 2016). As this trial is ongoing, the results of this paper are restricted to the first group of study participants. The study was composed of 4 parts: (1) the preintervention portion, where participants completed consent, an online survey, a semistructured interview, and installation of the study Facebook app; (2) the 6-week intervention; (3) the postintervention portion, where participants were asked to complete a postintervention survey, semistructured interview, and optional reflection group; and (4) the follow-up portion, where participants were asked to complete an online role transformation survey 6 weeks after completion of the intervention.

All participants provided written informed consent through the online survey page. Once participants met screening criteria and consented to the study online, they could proceed to the online survey preintervention portion of the study. The survey included questions on demographic information and required completion of 7 standardized scales (described below). Once we identified an applicant’s completion of the survey, one of the research team members conducted a semistructured telephone interview and guided the participant in installation of the study app.

The intervention comprised 2 major components: interaction within a closed Facebook online support group and posting of anonymous questions about caregiving to each participant’s Facebook News Feed through the study app. Participants interacted with other group members within the private Facebook online support group by posting and responding to each other’s comments. We facilitated group introductions and discussion through weekly prompts. These prompts requested participants to discuss emotional or informational support questions that the group chose to share with their social network (Facebook friends). We observed and moderated postings and participation. Moderation included sending reminder prompts to post information for participants who did not respond to the initial prompts to post information, answering technical and study activity questions, and monitoring for comments that could cause harm to the participants.

**Figure 1:**
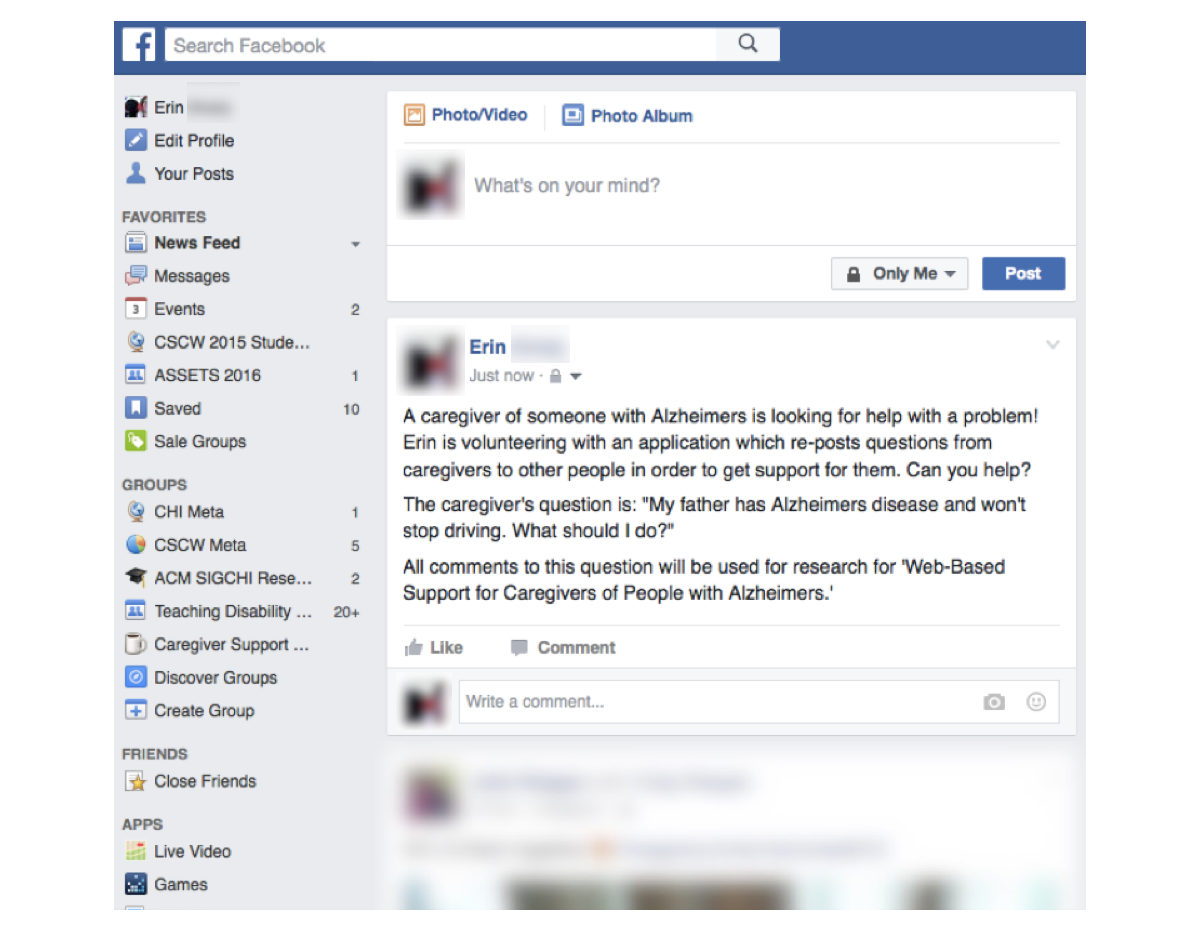
An example of a question from the caregiving group, which is automatically posted to the Facebook News Feed of each caregiver after screening by the research team.

Through the study app, selected questions were automatically posted to the Facebook News Feed of each participant so that his or her Facebook friends could review and post responses to the caregiver questions (Figure 1). These answers from Facebook friends were then reposted into the private Facebook group for group members to read as a way to leverage their impact on peer social support.

**Figure 2:**
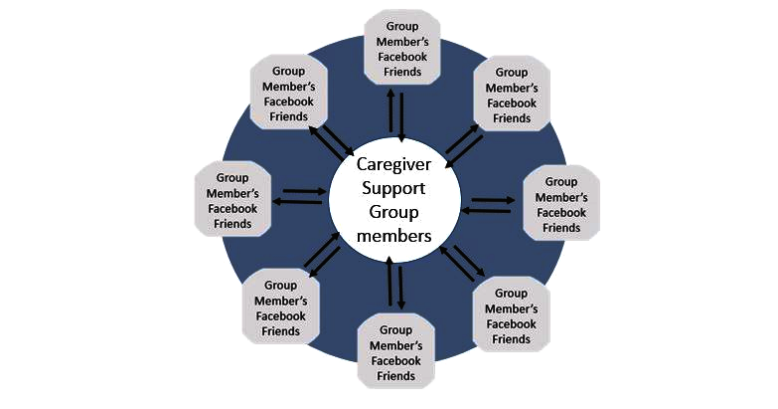
Leveraging multiple social networks for support increases the number of people available to answer questions.

By posting each question to the News Feed of each caregiver, we were able to expand the number of people available to provide support at any point. Figure 2 shows how the support network size is increased by using friends from multiple networks as potential question answerers. Prior work on social microvolunteering has shown that this use of multiple networks can approximate the speed of crowdsourcing in collecting answers to questions [39].

After finishing the 6-week intervention, participants were asked to complete the postsurvey and semistructured telephone interview. They were then offered the opportunity to participate in an optional reflection group and role transformation survey. Participants received compensation for their time and effort in the form of electronic gift cards.

### Data and Measures

We collected both quantitative and qualitative data at multiple time periods during the study. We gathered demographic variables, including age, sex, living arrangement, level of education, employment status, income, type of residential area, and relationship with the care recipient, through the preintervention survey. We also gathered qualitative data from preintervention and postintervention interviews and follow-up reflection group interviews. Additionally, comments were collected from the automatic posts made in each participant’s Facebook News Feed. These comments included those made by the participants and those made by their social network (Facebook friends) in response to posted caregiver questions.

The preintervention and postintervention online surveys included 7 standardized self-reported caregiving-related instruments: the Zarit Burden Interview Short Form (ZBI-12) [40], the Perceived Stress Scale-14 (PSS-14) [41], the Revised Scale for Caregiving Self-Efficacy [42], the Medical Outcomes Study (MOS) Social Support Survey [43], the Dementia Severity Rating Scale [44], the Neuropsychiatric Inventory Questionnaire [45], and the Facebook Intensity Scale (FBI) [46]. Below we describe 3 of the scales that capture caregiver needs, social support, and views on technology.

The ZBI-12 [40] measures caregivers’ perceived burdens of their caregiving roles. The measurement consists of 12 items in 2 main domains: personal strain and role strain. Each question asks about the frequency at which a caregiver experiences certain types of caregiving difficulties. It is scored in a 5-point Likert-type scale from 0 (never) to 4 (nearly always).

The MOS Social Support Survey measures the availability and frequency of different types of support [43]. The scale is composed of 19 items with 3 subsections—8 emotional and informational support sections, 4 tangible support sections, and 6 affectionate support sections—and 1 additional item. It is scored on a 5-point Likert scale from 1 (none of the time) to 5 (all the time).

The FBI is composed of 8 items about the level of familiarity with Facebook: 6 items were scored using a 5-point Likert scale from 1 (strongly disagree) to 5 (strongly agree) [46], and 2 open-ended questions assessed the user’s Facebook behavior regarding the total number of Facebook friends and the average hours per day of availability to use Facebook.

### Crowdsourcing Using Amazon Mechanical Turk

Through a separate study approved by the Indiana University Institutional Review Board (# 1609570045), we posted 2 of our caregiver questions to the Amazon Mechanical Turk (Amazon.com, Inc, Seattle, WA, USA). Amazon Mechanical Turk is a traditional crowdsourcing platform where a vast pool of workers can select tasks to complete for small payments [33]. Mechanical Turk is frequently used as an input to academic crowdsourcing systems, including those supporting emotional health [34] and those evaluating health literacy [47].

**Figure 3:**
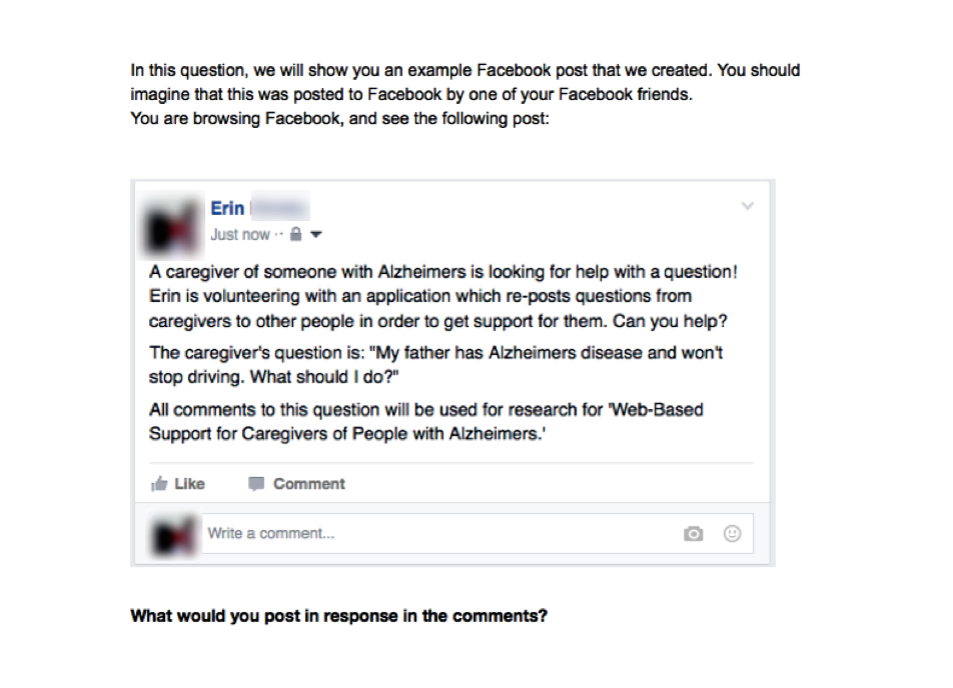
An example of a question posted on Amazon Mechanical Turk.

We posted our questions on Mechanical Turk with the goal of comparing these answers with the answers provided by our caregivers’ online social networks. We recruited Masters-level Turkers, who have met specific qualifications indicating their experience level with using the platform. Workers who accepted the task on Mechanical Turk were presented with an example question, shown in the same Facebook post format as was posted to the caregivers’ News Feed (Figure 3). They were asked how they would respond in the comments to that post.

The workers were paid US $3 for their response. While this is a higher payment rate than in many other Amazon Mechanical Turk studies [48], it is in line with worker expectations of payment on Amazon Mechanical Turk as a labor marketplace [49].

### Data Analysis

Survey results helped characterize participants through measures of caregiving stress, self-efficacy, familiarity with Facebook, care recipient symptoms, and illness severity. We audio recorded and transcribed participant interviews and then conducted a deductive thematic analysis of these transcriptions focused on participants’ access to support [50]. Interviews were open coded and themes were identified across participants and reported via prevalence.

We evaluated the Facebook friend (friendsourcing) answers to caregiving questions. Answers were reviewed by 2 content experts (DRB and DW) for acceptability, shared experiences, emotional support, and informational support. Agreement was obtained through a process of adjudication. Answers were described qualitatively. We used the same rating process for the crowdsourced answers obtained through Amazon Mechanical Turk. Acceptability was defined as an answer that, without further information or advice, would be unlikely to cause harm to a caregiver and care recipient, if it were to be read. We used definitions for “emotional support messages” and “informational support messages” from Wang et al [51]. Emotional support messages provide or show understanding, encouragement, affirmation, sympathy, or caring [51]. Informational support messages deliver advice, referrals, or knowledge [51].

## Results

### Participants

A total of 12 potential participants accessed the online study survey. Of these, 6 participants completed the online survey and semistructured telephone interview, and 4 of these 6 agreed to participate in the group activity portion of the study. Only 1 of the 6, P6, declined to participate, because she felt she did not need any extra support. Her MOS Social Support Survey results were consistent with this reasoning. Another participant, P5, declined, citing limited time as a result of caregiving obligations as the main reason for nonparticipation. She estimated her hours of caregiving at 148 hours per week, 128 hours longer than the next closest participant (Table 1). Participants’ ages ranged from 34 to 74 years, with a mean age of 58 years. Of the 6 participants, 5 had completed some college, with 2 participants having obtained advanced degrees. The hours of caregiving provided per week ranged from 16 to 148, with a mode of 20. Duration of caregiving ranged from 7 to 36 months, with a mean of 20 months. Half (n=3) of the caregivers were either a spouse or partner to the care recipient. All 5 of the care recipients (people with AD) were female. Participant ZBI-12 scores ranged from 12 to 30 (range 0-48) with a mean of 21.83 (SD 5.81) (Table 2).

**Table 1:** Demographic characteristics of persons with Alzheimer disease (AD) and their caregivers (study participants).

Characteristics		Participant					
		P1	P2	P3	P4	P5^a^	P6^a^
**Caregivers**							
	Sex	Male	Male	Female	Male	Female	Female
	Race/ethnicity	White	African American	White	White	White	Asian American
	Education	4-year college	4-year college	Master’s degree	2-year college	High school	Master’s degree
	Employment status	Retired	Retired	Full-time	Part-time	Retired	Full-time
	Marital status	Married	Not married	Married	Married	Divorced	Married
	Living arrangement	Living together	Living together	Living together	Living together	Living alone	Living together
	Living area	Suburban	Metro	Urban	Suburban	Suburban	Metro
	Self-reported health status	Good	Very good	Good	Good	Good	Good
	Age (years)	74	61	34	60	73	46
**Caregiving**							
	Duration of caregiving (months)	24	24	7	9	36	N/A^b^
	Caring time (hours/week)	20	N/A	16	16	148	N/A
**Person with AD characteristics**							
	Relation to caregiver	Wife	Partner	Mother	Wife	Mother	N/A
	Age (years)	74	55	65	65	93	N/A
	Sex	Female	Female	Female	Female	Female	N/A
	Race/ethnicity	White	White	White	White	White	N/A

^a^Did not participate in the group portion of the study.

^b^N/A: not available.

**Table 2:** Caregiver burden, social support, and technology use: baseline scores.

Measure		Participant						Mean (SD) score	
		P1	P2	P3	P4	P5^a^	P6^a^	n=6 (P1–P6)	n=4 (P1–P4)
Zarit Burden Interview Short Form (5-point scale; 12 items; range 0-48)		22	22	24	21	30	12	21.83 (5.81)	22.25 (1.26)
**Medical Outcomes Study Social Support Survey**									
	Emotional and informational support (8 items)	8	8	21	23	32	33		
	Tangible support (4 items)	16	4	12	12	10	16		
	Affectionate support (3 items)	12	15	15	12	11	12		
	Positive social interaction (3 items)	9	15	15	12	7	12		
	Additional item (1 item)	3	4	4	3	3	4		
	Total (5-point scale; 19 items; range 19-95)	48	46	67	62	63	77	60.50 (11.74)	55.75 (10.34)
**Facebook Intensity Scale**									
	Familiarity with Facebook (5-point scale; 6 items; range 6-30	12	6	7	23	6	16	11.67 (6.83)	12.00 (7.79)
	No. in Facebook network (approximate)	80	900	1000	40	400	100	420 (431.18)	505 (515.72)
	Available time to use Facebook (min/day)	20	60	180	0	120	15	65.83 (70.74)	65.00 (80.62)

^a^Did not participate in the group portion of the study.

### Deductive Thematic Analysis

#### Limitations of Current Support Resources

All 4 participants in the group portion of the study had access to some type of support for their caregiving, but these resources were limited in significant ways. All of our participants reported insufficient access to in-person support.

#### In-Person Support

Family was the most commonly referenced source of support, with all 4 participants describing family members who had provided them with emotional support. A total of 3 participants were geographically isolated from most of their family members, meaning that the burden of caregiving fell primarily on them. They also had to explicitly contact family members for emotional support. The caregivers were often the only family members with enough contextual information to make health decisions for their care recipient, meaning they could not rely on family members for informational support. Primary caregivers often instead provided support to those family members:

I think a lot of it is, I have a lot of family members that are in [state] that are not medical and sometimes don’t understand what is going on with her. They ask me a lot of questions...I just want to keep her comfortable and they don’t always understand that. I think that’s hard for me to justify all of my actions with her.

P3

Many participants experienced a change in their ability to leave their home as a result of their caregiving, which meant they had less access to social or emotional support from friends in workplace or recreational settings. Indeed, 2 participants had cut back or left their jobs to focus on caregiving full-time, while others gave up existing hobbies or social activities, so as not to leave their care recipient alone. These changes may have led to a smaller network of support immediately available to them.

All 4 caregivers relied on their loved one’s doctors as a primary resource for informational support.

The doctors observed macro-level changes in the patients’ behavior and health, rather than day-to-day issues. Support from physicians was infrequently available. For example, P4 specified that he interacted with his wife’s doctor only once every 3 months. Some caregivers also used medical professionals as a source of emotional support, and 2 participants sought formal emotional support through therapy. As with informational support, professional emotional support was highly valued by the caregivers who accessed it. However, long intervals occurred between visits, and caregivers expressed a wish to have access to additional emotional support.

#### Online Support

To join our study, participants were required to have an active Facebook account with at least 40 friends, meaning that our participants were likely to have significant technological experience. All participants reported having both computers and smartphones through which they accessed the Internet. Despite this baseline level of technology and social media use, we found a variety of use patterns and technology acceptance among our participants.

Two caregivers, P1 and P4, had small networks on Facebook (80 and 40 estimated friends, respectively; Table 2). Both primarily used the sites to catch up with friends and family. While P4 had not previously used Facebook to access emotional support, P1 shared how his family used Facebook to encourage him during his caregiving:

One day my daughter posted on Facebook what a great job I was doing taking care of her mom and so on.

P1

The other 2 participants, P2 and P3, had large networks on Facebook (900 and 1000 estimated friends, respectively; Table 2) and used the site both to socialize and to access news or information about AD. P3 described how she valued Facebook as a resource for her friendships and for learning:

I look up sports there [on Facebook] and I follow three different Alzheimer’s things. I try to mix work there so that way I will look at it. It’s like a one-stop news place for me. Equal half for friends and information.

P3

While all participants except P4 used other social network sites, like Twitter or Pinterest, all participants reported that Facebook was the site that they used with the highest frequency.

Caregivers used a variety of online resources to access informational support. Some of that information was acquired passively, by following or subscribing to updates from AD organizations via Facebook or email. Others sought information actively by researching specific informational questions. While participants used online forums to research information support questions, none had posted on online forums or discussion groups related to caregiving themselves. P4 reported that he had browsed one of these forums for information, but had not yet posted his own questions:

I read them. I have thought of posting on it. I’m still new on it. So I’m kind of reading other experiences, because there is so much good information in there. For example, a lady posted that her dad keeps wandering away from the house or another posts about how their mom does not sleep at night. How to handle that. I read that to see their responses. It’s been very helpful. Yes, reading about other people’s experiences [is] very helpful.

P4

I’m not sure. I tend to be a private person. I would not be comfortable opening up with people [I] don’t know with the exception of Facebook. There is this thing about the Internet where I may never meet that person.

P1

Our participants’ responses indicate a need for additional support, as well as a familiarity with Facebook that might make it a more appealing source of support than anonymous forums or crowdsourcing platforms.

### Activity During Facebook Online Support Group

During the online Facebook group portion of the study, we posted 4 online prompts (Table 3). There were a total of 12 replies (postings) by group members in response to our prompts. The group participants had 20 spontaneous voluntary posts or replies to other members of the group.

**Table 3:** Summary of online activity for the whole group and by individual participants.

Type of activity			Group totals	Individual participants			
				P1	P2	P3	P4
**Group activities**							
	No. of group participants (total)		4				
	No. of posted questions by the research team (total)		4				
	**No. of activities in the group (total)**		32				
		Replies and posts responding to requests from the research team	12				
		Unprompted postings and replies from group members	20				
**Facebook crowds activities**							
	No. of questions pushed to Facebook friends (total)		3				
	No. of answers from Facebook friends (total)		44	6	19	11	11
	**No. of answers from Facebook friends (mean)**			2	6.33	3.67	3.67
		No. of answers to first question		1	11	6	2
		No. of answers to second question		2	4	1	5
		No. of answers to third question		3	4	4	4
	Reported no. of Facebook friends			80	900	1000	40

### Friendsourcing Results

We pushed 3 caregiver questions to the participants’ Facebook News Feed, allowing participants’ Facebook friends to see and reply to the caregiver questions. The total number of Facebook friend responses was 44 (Table 3). The range of total number of Facebook friend responses per participant varied between 6 and 19.

Friendsourcing and crowdsourcing answers had high and similar rates of acceptability, as judged by content experts: 90% (27/30) and 100% (45/45), respectively (Table 4). This study lacked the power to draw quantitative conclusions from these data. Friendsourced answers contain shared experiences at a higher rate than Amazon Mechanical Turk answers: 67% (20/30) and 9% (4/45), respectively. Rates of emotional support and informational support messages present in friendsourcing and crowdsourcing answers were similar, and appeared more dependent on the type of question asked rather than the group answering the question.

**Table 4:** Caregiver questions answered through friendsourcing and crowdsourcing (Amazon Mechanical Turk).

Type of answers		Q1^a^	Q2^b^	Total, n (%)
**Friendsourcing answers**				
	Total number of answers	19	11	
	Acceptable answers, n (%)	16 (84)	11 (100)	27/30 (90)
	Shared experiences, n (%)	12 (63)	8 (73)	20/30 (67)
	Combined (informational + emotional support), n (%)	5 (26)	5 (45)	
	Informational support, n (%)	19 (100)	7 (63)	
	Emotional support, n (%)	5 (26)	9 (82)	
**Crowdsourcing answers**				
	Total number of answers	20	25	
	Acceptable answers, n (%)	20 (100)	25 (100)	45/45 (100)
	Shared experiences, n (%)	3 (15)	1 (4)	4/45 (9)
	Combined (informational + emotional support), n (%)	6 (30)	15 (60)	
	Informational support, n (%)	19 (95)	21 (84)	
	Emotional support, n (%)	7 (35)	19 (76)	

^a^Q1 was “My father has Alzheimer’s disease and won’t stop driving. What should I do?”

^b^Q2 was “It is very hard for me to share my personal feelings about my struggles with my mother’s Alzheimer’s so when people ask about how my mother’s doing, I either minimize her symptoms or just unload on them. How can I explore my own feelings better without having to talk to someone so that I can better communicate about my mother’s battle with Alzheimer’s? I would love to be an advocate for Alzheimer’s awareness without turning people off to talking about it.”

### Effects of the Intervention

Our small sample prevented the use of parametric statistics; thus, we used Wilcoxon signed rank tests to compare preintervention and postintervention measures. This nonparametric method considers scores as ranks to measure changes between 2 periods. We calculated both medians and means. None of the pretest-posttest comparisons showed a statistically significant difference (Table 5). However, caregiver burden (measured by ZBI-12) showed a trend toward improvement in overall caregiver burden. There were trends toward an increase in emotional and informational support, tangible support, and total social support, but these results were not statistically significant. FBI showed a near doubling of the level of familiarity with Facebook. The number of Facebook friends and time available to use Facebook also increased. The median and mean scores of PSS-14 frequencies (having emotional problems in the last month) decreased, although the change was not statistically significant. The Revised Scale for Caregiving Self-Efficacy, a measure of confidence regarding caring activities, showed a trend toward improved confidence.

**Table 5:** Comparisons of preintervention and postintervention caregiver data.

Scale		Pre-scores (A), median (mean) (n=6)	Pre-scores (B), median (mean) (n=4)	Post-scores (C), median (mean) (n=4)	Difference (C–B)	
					*z* Score	*P* value
Zarit Burden Interview Short Form		22.00 (21.83)	22 (22.25)	18.00 (18.75)	–1.29	.20
**Medical Outcomes Study Social Support Survey**						
	Emotional and informational support	22 (20.83)	14.5 (15)	22.5 (21.25)	–1.63	.10
	Tangible support	12 (11.67)	12 (11)	15 (13.5)	–1.07	.29
	Affectionate support	12 (12.83)	13.5 (13.5)	12 (10)	–1.07	.29
	Positive social interaction	12 (11.67)	13.5 (12.75)	9 (9)	–1.13	.26
	Additional item	3.5 (3.5)	3.5 (3.5)	3 (3)	–0.54	.59
	Total scores	62.5 (60.5)	55 (55.75)	62 (56.75)	–0.37	.72
Perceived Stress Scale-14		31 (31.67)	31 (31.25)	22.5 (22.75)	–1.83	.07
**Revised Scale for Caregiving Self-Efficacy**						
	Self-efficacy for obtaining respite	250 (213.33)	250 (197.5)	230 (215)	–0.54	.59
	Self-efficacy for responding to disruptive patient behaviors	445 (445)	440 (440)	430 (397.5)	–0.92	.36
	Self-efficacy for controlling upsetting thoughts about caregiving	335 (335)	340 (315)	345 (342.5)	–0.55	.58
	Total scores	975 (993.33)	975 (952.5)	1040 (955)	0.00	>.99
**Facebook Intensity Scale**						
	Familiarity with Facebook	9.5 (11.67)	9.5 (12)	17.55 (21.55)	–1.83	.07
	Approximate no. in Facebook network	250 (420)	490 (505)	620 (585)	–1.60	.11
	Time to use Facebook (min/day)	40 (65.83)	40 (65)	60 (53.75)	0.00	>.99

## Discussion

### Feasibility of the Intervention

We evaluated the feasibility of soliciting acceptable answers to informational and emotional questions through the Facebook News Feeds of caregivers. Based on participation and qualitative feedback from participants, our study found friendsourcing to be a feasible Web-based intervention for AD caregivers. Our online support group and app successfully facilitated “pushing” caregiver questions to the Facebook News Feed of participants, allowing their Facebook friends to see and answer these questions. We compared friendsourcing versus crowdsourcing answers to caregiver questions. Both provided acceptable answers as judged by content experts, as well as similar rates of informational and emotional support messages. However, we consistently found that friendsourcing provided significantly higher rates of shared experiences as compared with crowdsourcing.

While this approach was feasible to collect acceptable answers in the short term, sustainability is an important concern for social-microvolunteering systems [39]. For this system to succeed in a full deployment, it would require a reliable set of answerers, and the quality of responses would need to be maintained despite the potential for answerer fatigue. This needs to be explored further in future studies.

Central to obtaining support and responding to demands for coping is the shared experience of group membership. Shared experience has been identified as the basis for engagement in peer support groups, because peers can provide a level of support that may be unavailable through natural supports like family and friends [52]. We designed our Web-based support intervention to include friendsourcing rather than crowdsourcing, for reasons of greater financial affordability and greater potential for shared experiences.

### Limitations

Low sample size was a drawback of this study and limits the generalizability of our results. Our study was too small to determine the effectiveness of the intervention on caregiver outcomes. However, we noted that data trended toward an improvement in caregiver self-efficacy, sense of support, and perceived stress.

The generalizability of the populations who provided the friendsourced and crowdsourced answers may also be limited. Members of crowdsourcing platforms, specifically those on Mechanical Turk, are likely to be more educated than the average US population [53]. Any of a user’s Facebook friends are most likely to be the same age as the user, demonstrating the homophily of networks around age [54]. As a result, a caregiver’s network may be composed of more adults with an age demographic more likely to deal with AD in their spouses or parents. Users of Mechanical Turk have traditionally skewed younger [55,56], and recent estimates found that 88% of workers were 49 years or younger [55]. Thus, these workers may not have as much experience with AD.

The duration of this study was relatively brief at 6 weeks. It is unclear whether a longer trial of an online support group and friendsourced answers would provide a greater benefit for AD caregivers. However, Rains and Young’s 2009 meta-analysis of peer support group outcomes suggest a longer trial would provide greater benefits to caregivers [57].

### Friendsourcing Versus Crowdsourcing

To the best of our knowledge, our study is the first to examine friendsourcing and crowdsourcing as a tool to support caregivers of people with AD. Studies have looked at medical and behavioral science applications of crowdsourcing. Yu et al found that medical pictogram responses from the crowdsourcing platform Mechanical Turk were comparable with responses solicited in laboratory studies [47]. They concluded that the platform could be used as a low-cost alternative to traditional experimental studies. Other work has found similar results in using Mechanical Turk as a venue to collect survey responses [58], run behavioral experiments [59], collect natural language samples [60], and build more complex algorithms that leverage human computation [61].

### Social Media for Caregivers and Emotional Support

With the changes brought by Web 2.0, social media became another means of collecting health care information [62]. Facebook remains the most widely used social media platform among the 73% of online adults who use a social networking website [63]. A few of the benefits of using social media as reported by adults seeking health care information are interactions with others with the same condition, increased availability of information, and emotional support [52,64]. Tailoring information and experience to suit personal needs is another benefit that draws users [52]. Caregiver use of the Internet and social media is greater than that of noncaregivers. In a survey of caregivers’ online health behaviors, caregivers used the Internet to obtain health information more than noncaregivers, at 72% and 50%, respectively [65]. In the same survey, 52% of caregivers participated in online social activity as compared with 33% of noncaregivers. [65].

Online peer support groups provide caregivers with a common platform to address emotional needs [66,67]. In addition to being able to share personal problems and stories and to seek advice in an easy-to-access venue, caregivers also feel empowered by the group experience that affords intimacy and bonding [67]. Colvin et al reported that the anonymity afforded by Internet-based social support made users more comfortable with using online support groups as compared with their face-to-face alternatives [68]. Also, the asynchronous and immediate availability of information or answers to questions accommodate caregiving needs, such as not having to leave the care recipient and finding answers faster.

### Ethical Considerations

The collaborative nature of social media leads to information collated from disparate sources, which could be inconsistent with a health care professional’s knowledge and opinions. Peer-to-peer communication does not ensure the regulation and validity of information. Users often report that they crosschecked information online or waited for consensus to develop in a group before they regard a piece of information as credible [69]. The task of processing large volumes of online information and deriving insights falls on the users. Medical providers often express concerns that inaccurate health information will be shared in online communities [70]. Most online communities address this issue through online moderation.

For many online communities, moderation is limited to policing of requests for answers to clinical questions. In these situations, moderators will typically close a post and indicate to a user that he or she should see his or her doctor to address this question or a “SeeDoc” thread [29]. Downsides to this approach include missed opportunities for sharing of experiences, informational support, and emotional support. Some authors argue that peers offer an expertise distinct from the medical expertise of health care professionals [71].

In unmoderated online peer support groups, nonparticipation or reading-without-posting behavior has been identified as a drawback that reduces overall group interaction and the development of mutual aid [30]. With our study design, we showed that an online social media peer support group was feasible. Moderation in our study reduced reading-without-posting behavior.

### Future Research

Work presented here included the limited analysis of the first group of our study cohort. Following completion of the project (2 more groups), we hope to gain further insights into how member interaction is influenced by friendsourced answers. We are also interested in learning how caregiver outcomes may be modified by an online support group and friendsourced support.

A similar study with a larger sample size and longer intervention is needed to determine the effectiveness of the intervention on caregiver outcomes and the overall sustainability of the intervention. Determination of effectiveness would also allow for comparisons with other existing caregiver interventions. We believe there would be value in adding caregiver mental health outcomes to future studies, as a modification of these outcomes by either crowdsourcing or friendsourcing could have wide implications. Additionally, it would be helpful to redesign the study to allow for a systematic comparison of support received from crowdsourcing, friendsourcing, and interactions between group members. Further study of the extended networks and the impact of ad hoc supportive members is also needed. In future work, we plan to complete a qualitative analysis of the open-ended questions answered in the preintervention and postintervention study interviews.

Friendsourcing and crowdsourcing offer new opportunities for caregivers of people with AD to receive informational and emotional support; however, concerns still exist around delivery of inaccurate health information. More work needs to be done to assess the quality of information received through these platforms. The growth of social media and online health community participation in the United States make the need for this work even more important, as people are likely to continue to use these online venues.

### Conclusions

We found this asynchronous, online, closed group on Facebook to be generally acceptable by the peer support group studied as a means to deliver support to AD caregivers. Implications could be wide reaching if larger studies find a significant impact on caregiver outcomes, as a similar intervention could be applied to caregivers of other diseases, such as cancer, serious mental illness, and developmental disabilities.

Both friendsourcing and crowdsourcing displayed potential as novel delivery methods of emotional and informational support to AD caregivers. Friendsourced answers demonstrated higher rates of shared experiences, which suggests that friendsourcing may be superior.

## Abbreviations

AD: Alzheimer disease
FBI: Facebook Intensity Scale
MOS: Medical Outcomes Study
PSS-14: Perceived Stress Scale-14
ZBI-12: Zarit Burden Interview Short Form
